# Meetings

**Published:** 1892-03

**Authors:** 


### Bristol Medico-Chirurgical Society.

*January 13th*, 1892.

Mr. F. R. Cross, President, in the Chair.

The following card specimens were shown:—(1) By Mr. C. A. Morton: (*a*) Primary necrosis of pubes, with secondary destructive disease of the hip-joint and psoas abscess, from a baby; (*b*) malignant stricture of rectum; and (*c*) large tubercular growth in finger, resembling a tumour. (2) By Dr. Rogers: a mass of uric acid removed from an abscess in a gouty patient.

Dr. Harrison and Dr. Waldo gave a demonstration on some interesting skin cases. Amongst these were: (1) Urticaria pigmentosa in a girl aged 16; (2) Erythema induratum in a woman, shown by Dr. Harrison: and (1) diffuse favus of the scalp; (2) kerion, following tinea tonsurans; (3) lupus of face much improved by cauterisation with acid nitrate of mercury; (4) diffuse pigmentation following lichen verrucosa, by Dr. Waldo.

Dr. Barrett Roué showed a case of eczema sqamosum.

Mr. C. H. Walker showed a patient with retinal hemorrhages.

Dr. Ewen Maclean exhibited an instrument for the accurate estimation of the knee-jerk. (See p. 20.)

Dr. Watson Williams read a paper on auscultatory percussion as a means of gaining accurate knowledge as to the size of the heart during life. Diagrams were shown in which the cardiac dulness had been mapped out, and the area thus obtained verified by *post mortem* examination. It was maintained that this method gave much better results than those hitherto employed.

Dr. Michell Clarke showed three specimens of mitral, complicated by tricuspid, stenosis, and pointed out how this might be clinically recognised by the early occurrence of ascites and enlargement of the liver, &c. Microscopic specimens of the diffused hepatic cirrhosis met with in these cases were shown. A saccular aneurism of the aortic arch was also shown, of which the only symptom during life was paralysis of the left vocal cord.

Mr. Ewens exhibited (1) a boy who had suffered from necrosis of right tibia, partial dislocation backwards at knee-joint, and displacement of head of femur on to dorsum ilii. The knee-joint was excised, the neck of the femur subcutaneously divided, and a useful limb resulted; (2) A girl who had been cured of double genu valgum by Macewen's operation on the thigh, and removal of wedge-shaped piece of bone from the tibia.

---

*February 10th*, 1892.

Mr. F. R. Cross, President, in the Chair.

The President announced the death of Dr. Burder, one of the Past Presidents of the Society, which took place on February 6th, and spoke of the deceased's connection with the General Hospital and Medical School, and of his skill as a meteorologist. A resolution was passed expressing sympathy with Dr. Burder's friends, and recording the loss the Society had sustained by his death.

Mr. C. A. Morton exhibited several specimens of tubercular hip-joint disease, together with microscopic sections.

Mr. F. P. Lansdown showed a specimen of bullet-wound of the external iliac artery from a boy who had been accidentally shot.

Dr. Markham Skerritt read notes on a case of chyluria. The patient, a lady, first suffered from it in the West Indies, and during the last seven years had had repeated attacks. A filaria was found in the urine, which fluid was milky, with a soft clot. Dr. Skerritt also showed a large sarcoma growing from the ovary of a woman, aged 23. The mass filled the lower part of the abdomen, and there were deposits of pedunculated growths on the abdominal walls and intestines, evidently arising from contact with the original tumour.

Mr. C. H. Walker showed a specimen, with photograph, of an eyeball with a calcareous, dislocated lens, which had caused sympathetic irritation in the opposite eye. The original injury was a gun-shot wound thirty-four years ago; the shot had entered the lower part of the ciliary region and passed out near the entrance of optic nerve, causing complete blindness. Recovery quickly followed removal of this eye.

Dr. B. M. Rogers read a paper on Micro-Organisms and Immunity from Disease. He divided the diseases which were generally supposed to be due to germs into two classes (1): those which were obviously due to this cause; and (2) the acute specific fevers, the etiology of which he considered not yet established. He pointed out that in these latter, one attack conferred protection; whereas in the first class, second or numerous attacks were common. The evidence of various authorities was quoted as to second attacks in diphtheria, erysipelas, cholera, &c. The belief was expressed that protective vaccinations with attenuated virus would not produce permanent immunity. Dr. Rogers maintained that as the diseases *certainly* caused by germs gave no protection from subsequent attacks, in those in which a second attack is rare the cause must not be sought for amongst micro-organisms or their products.

---

*March 9th*, 1892.

Mr. F. R. Cross, President, in the Chair.

Dr. Ogilvy showed a boy with an eyelash in the anterior chamber, and a small (probably sebaceous) tumour growing from its root, which was situated at the corneal margin. It was carried into the eye with a piece of wire, which caused a traumatic cataract.

Dr. Prowse read notes of a case of adherent and thickened pericardium, and showed the specimen, which was from a man aged 25, who had suffered for some months before his death from shortness of breath, dyspnœa on exertion, and œdema of the limbs. He was tapped both in the pleuræ and abdomen on several occasions and a large quantity of fluid removed, with temporary relief. The pericardium was found, *post mortem*, to be very thick, almost cartilaginous in places, and firmly adherent to the heart, œsophagus, and trachea. There was evidence of fibroid change in other organs (liver, spleen, &c.).

Dr. Barrett Roué described a case of diphtheria in a child aged 19 months, following measles, in whom intubation of the larynx was performed for urgent dyspnœa. Recovery took place. The comparative merits of tracheotomy and intubation were discussed and statistics quoted.

Mr. Morton showed drawings and specimens of five cases of tubercle of the choroid. He called attention to the microscopic structure of these growths, the ophthalmoscopic appearances and the diagnosis from patches of choroidal atrophy, and laid stress on the frequency of this condition in tubercular meningitis.

He also showed two specimens, with microscopic sections and drawings, of tubercular ulcers of the tongue. He pointed out the characteristics by which these ulcers could be distinguished from epithelioma, and discussed the methods by which the tongue became inoculated with the tubercle bacillus.

---

At a Meeting of the Committee, held on March 11th, the following Resolution was unanimously passed:

> "The Members of the Committee of the Bristol Medico-Chirurgical Society accept with much regret Mr. J. Greig Smith's resignation of the post of Editor of the Society's *Journal*, an office which he has held with such distinguished ability since the foundation of the *Journal* in 1883. They desire to express the great obligation which the Society is under to him for the leading part he took in the creation of the *Journal*, and for the time and ability which he has expended in its conduct and development."

G. Munro Smith, *Hon. Sec.*

